# Rim Augmentation to Facilitate Gore Distal Disc Cupping for a Large Atrial Septal Defect Closure

**DOI:** 10.1016/j.shj.2026.101116

**Published:** 2026-08-12

**Authors:** Alice Moroni, Kitae Kim, Abdulaziz Al-Shaibi, Prasham Dave, Neil Fam, Sami Alnasser

**Affiliations:** Structural Heart Program, St Michael’s Hospital, University of Toronto, Toronto, Ontario, Canada

**Keywords:** Atrial septal defect, GORE CARDIOFORM occluder

Transcatheter atrial septal defect (ASD) closure using the GORE CARDIOFORM ASD Occluder has demonstrated favorable outcomes and no reported cases of erosion, making it an attractive option for closure of large defects with deficient septal rims.^1^ During deployment, firm traction of the left atrial disc against the septal tissue (“cupping”) is an essential step that ensures appropriate positioning of the occluder discs across the septum. However, in large defects with minimal rims—particularly the superior rim—adequate cupping may not be feasible, leading to nonseptal device positioning, inadequate rim engagement, and potential need for surgical repair as an alternative closure strategy.

A 32-year-old woman with a large secundum ASD (30-mm stop-flow diameter), deficient anterosuperior rims, and right ventricular enlargement (Figure 1A and B) underwent closure. Multiple deployment attempts of a 48-mm Gore ASD device with varying delivery trajectories failed to achieve adequate cupping of the Gore device, resulting in the device slipping off the superior rim (Figure 1C) or failing to engage the inferior-posterior rim (Figure 1D). Given the patient’s young age and deficient aortic rim, the GORE CARDIOFORM ASD Occluder was preferred over the Amplatzer Septal Occluder. In addition, its softer design may facilitate future trans-septal access should left-sided transcatheter interventions become necessary. Accordingly, additional vascular access was obtained, and a 34-mm Amplatzer Sizing Balloon was advanced across the interatrial septum. Partial balloon inflation across the defect augmented the superior rim, enabling effective cupping of the distal disc (Figure 1E and F). The device was then deployed with simultaneous balloon deflation, allowing secure engagement of the septal rims (Figure 1G). Initial device waist constraint related to deployment around the balloon resolved after balloon removal, and final device locking confirmed stable positioning without residual shunt (Video 1). Fluoroscopy time and total procedural time were 35 and 104 minutes, respectively.

**Figure 1:**
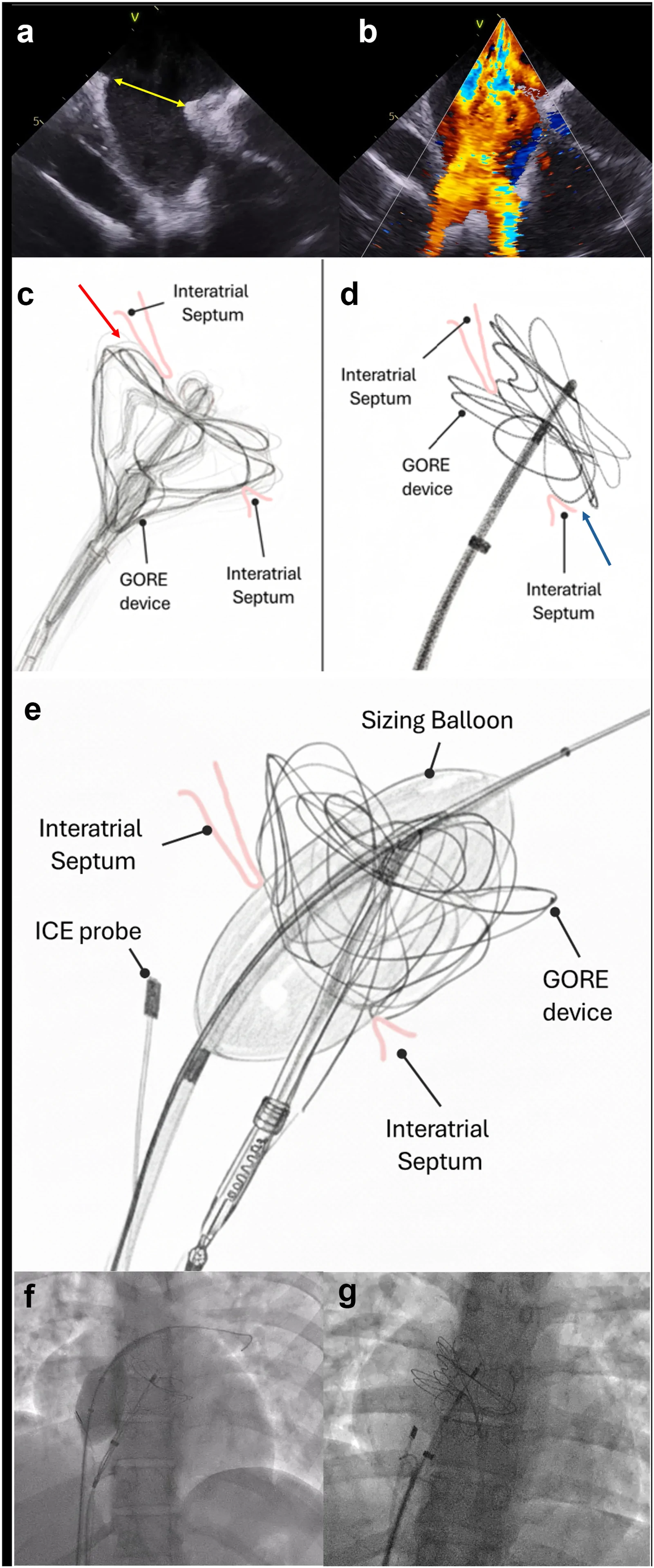
**(a and b)** ICE views of the ASD. **(c)** Failed distal disc cupping with disc slippage off the superior rim (red arrow). **(d)** Incomplete rim engagement (blue arrow). **(e and f)** Partial inflation of a sizing balloon to augment the superior rim, enabling effective cupping. **(g)** Successful deployment of a 48-mm occluder. Abbreviations: ASD, atrial septal defect; ICE, intracardiac echocardiography.

Balloon-assisted techniques have previously been described to facilitate the coaxial deployment of Amplatzer devices.^2^ In this case, balloon assistance uniquely enhanced the anterosuperior rim, enabling effective distal disc cupping and successful Gore device deployment in a large ASD with limited septal tissue, thereby highlighting a useful adjunctive strategy in challenging anatomies.

## Consent Statement

Patient consented to sharing clinical details.

## Funding

The authors have no funding to report.

## Disclosure Statement

The authors report no conflict of interest.

## Supplementary material

Video 1 Fluoroscopic and ICE views of the balloon-assisted rim enhancement maneuver. Abbreviations: ICE, intracardiac echocardiography.

## References

[1] Qureshi A.M., Sommer R.J., Morgan G. (2024). Long-term results of the atrial septal defect occluder ASSURED trial for combined pivotal/continued access cohorts. JACC Cardiovasc Interv.

[2] Dalvi B.V., Pinto R.J., Gupta A. (2005). New technique for device closure of large atrial septal defects. Catheter Cardiovasc Interv.

